# Efficacy and safety of different radiotherapy doses in neoadjuvant chemoradiotherapy in patients with locally advanced rectal cancer: A retrospective study

**DOI:** 10.3389/fonc.2023.1119323

**Published:** 2023-02-21

**Authors:** Yuyan Xu, Haizhou Zou, Zhenyong Shao, Xuebang Zhang, XiaoLin Ren, Huijuan He, Dahai Zhang, Dexi Du, Changlin Zou

**Affiliations:** ^1^ Department of Radiotherapy, The First Affiliated Hospital of Wenzhou Medical University, Wenzhou, China; ^2^ Department of Oncology, Wenzhou Hospital of Traditional Chinese Medicine, Wenzhou, China; ^3^ Department of Radiotherapy, Quzhou People’s Hospital, Quzhou, China; ^4^ Department of Radiotherapy, Dongyang People’s Hospital, Jinhua, China; ^5^ Department of Radiotherapy Oncology, Lishui Central Hospital, Lishui, China

**Keywords:** locally advanced rectal cancer, radiotherapy dose, intensity-modulated radiation therapy, neoadjuvant chemoradiotherapy, adverse reactions

## Abstract

**Background:**

This study aims to compare the efficacy and safety of neoadjuvant chemoradiotherapy (nCRT) with different radiotherapy doses (45Gy and 50.4Gy) in patients with locally advanced rectal cancer (LARC).

**Methods:**

Herein, 120 patients with LARC were retrospectively enrolled between January 2016 and June 2021. All patients underwent two courses of induction chemotherapy (XELOX), chemoradiotherapy, and total mesorectum excision (TME). A total of 72 patients received a radiotherapy dose of 50.4 Gy, while 48 patients received a dose of 45 Gy. Surgery was then performed within 5-12 weeks following nCRT.

**Results:**

There was no statistically significant difference between the baseline characteristics of the two groups. The rate of good pathological response in the 50.4Gy group was 59.72% (43/72), while in the 45Gy group achieved 64.58% (31/48) (P>0.05). The disease control rate (DCR) in the 50.4Gy group was 88.89% (64/72), compared to 89.58% (43/48) in the 45Gy group (P>0.05). The incidence of adverse reactions for radioactive proctitis, myelosuppression, and intestinal obstruction or perforation differed significantly between the two groups (P<0.05). The anal retention rate in the 50.4Gy group was significantly higher in contrast to the 45Gy group (P<0.05).

**Conclusions:**

Patients receiving a radiotherapy dose of 50.4Gy have a better anal retention rate but also a higher incidence of adverse events such as radioactive proctitis, myelosuppression, and intestinal obstruction or perforation, and a comparable prognosis to patients treated with a radiotherapy dose of 45Gy.

## Introduction

Colorectal cancer incidence and mortality ranked third and second overall in 2020, according to cancer statistics (1). It is common for patients with rectal cancer to be asymptomatic in the early stages, so many patients are already in an advanced stage upon diagnosis. Neoadjuvant chemoradiotherapy (nCRT) plays an important role for patients with locally advanced rectal cancer (LARC), and clinical trials like the CAO/ARO/AIO-94 Study, the Swedish Trial, and the CAO/ARO/AIO-04 Study have demonstrated its effectiveness in LARC patients (2–5). Neoadjuvant chemoradiotherapy combined with total mesorectum excision (TME) is the first-line treatment for LARC patients, and while it can reduce tumor burden, induce downstaging, and improve the local control rate, it does not improve the overall survival (OS) (4, 6–8).

In recent years, the development of total neoadjuvant therapy (TNT) has also provided new options for LARC patients. CAO/ARO/AIO-12 Trial assessed the outcomes of 311 LARC patients treated with chemotherapy plus chemoradiotherapy (CRT) plus TME or CRT plus chemotherapy plus TME, and showed that if organ preservation is a priority, then TNT with consolidation chemotherapy (CNCT) after CRT is the preferred modality and this trial provides important evidence for the clinical use of TNT and has influenced the concept of organ preservation (9). RAPIDO trial looked at the efficacy of preoperative short-course radiotherapy (SCRT) plus nCRT compared to preoperative concurrent chemoradiotherapy (CCRT) and concluded that SCRT combined with nCRT reduced the probability of treatment failure in rectal cancer compared to standard treatment (10). The findings suggest that preoperative chemotherapy may be more effective than adjuvant chemotherapy and that this treatment modality may become the new standard of care for high-risk LARC (10). OPRA trial analyzed the outcomes of 324 LARC patients treated with induction chemotherapy (INCT) followed by CRT or CRT followed by CNCT, and concluded that half of the patients who received neoadjuvant treatment achieved organ preservation with no significant impairment in survival compared to previous controls who received radiotherapy, TME, and post-operative chemotherapy (11).

Notably, outcomes of nCRT vary widely among LARC patients, with more than one-third of patients experiencing recurrent or metastatic diseases (12). Moreover, some patients can achieve pathological complete remission (pCR) while others barely respond to nCRT. The proportion of patients who achieve pCR after nCRT is usually used as a reliable indicator of treatment response, Roh et al. reported that about 10-30% of patients achieved pCR (13), while Sanghera et al. concluded that 42% reached pCR (14) after nCRT. There is a strong correlation between clinical stage, tumor differentiation, and treatment regimens (6, 15, 16) in determining the outcomes of patients. Given that patients respond differently to nCRT, more research is needed to identify the most effective follow-up treatments.

Generally, surgery, chemotherapy, and radiotherapy are all crucial treatments for LARC, and each of these methods has made significant progress in recent years (17–23). The National Comprehensive Cancer Network (NCCN) guidelines and European Society for Medical Oncology (ESMO) guidelines recommend a total dose of 45-50.4Gy delivered in 25-28 fractions (24). In clinical practice, both radiotherapy doses are frequently used, but studies assessing their efficacy and safety are scarce. More studies focus on radiotherapy dose intensification versus conventional fractionation (25–28). Higher radiotherapy doses are known to be associated with improved efficacy but are also associated with an increased incidence of adverse events. A comparative analysis of radiotherapy doses of 45 Gy and 50.4 Gy was performed in the present study. Patients were divided into the 45Gy group and the 50.4Gy group according to the radiotherapy dose. Notably, pathological responses, imaging assessments, anal retention rate, local control, adverse reactions, and survival were analyzed across the two groups (45Gy and 50.4Gy).

## Materials and methods

### Patient selection

**Figure 1:**
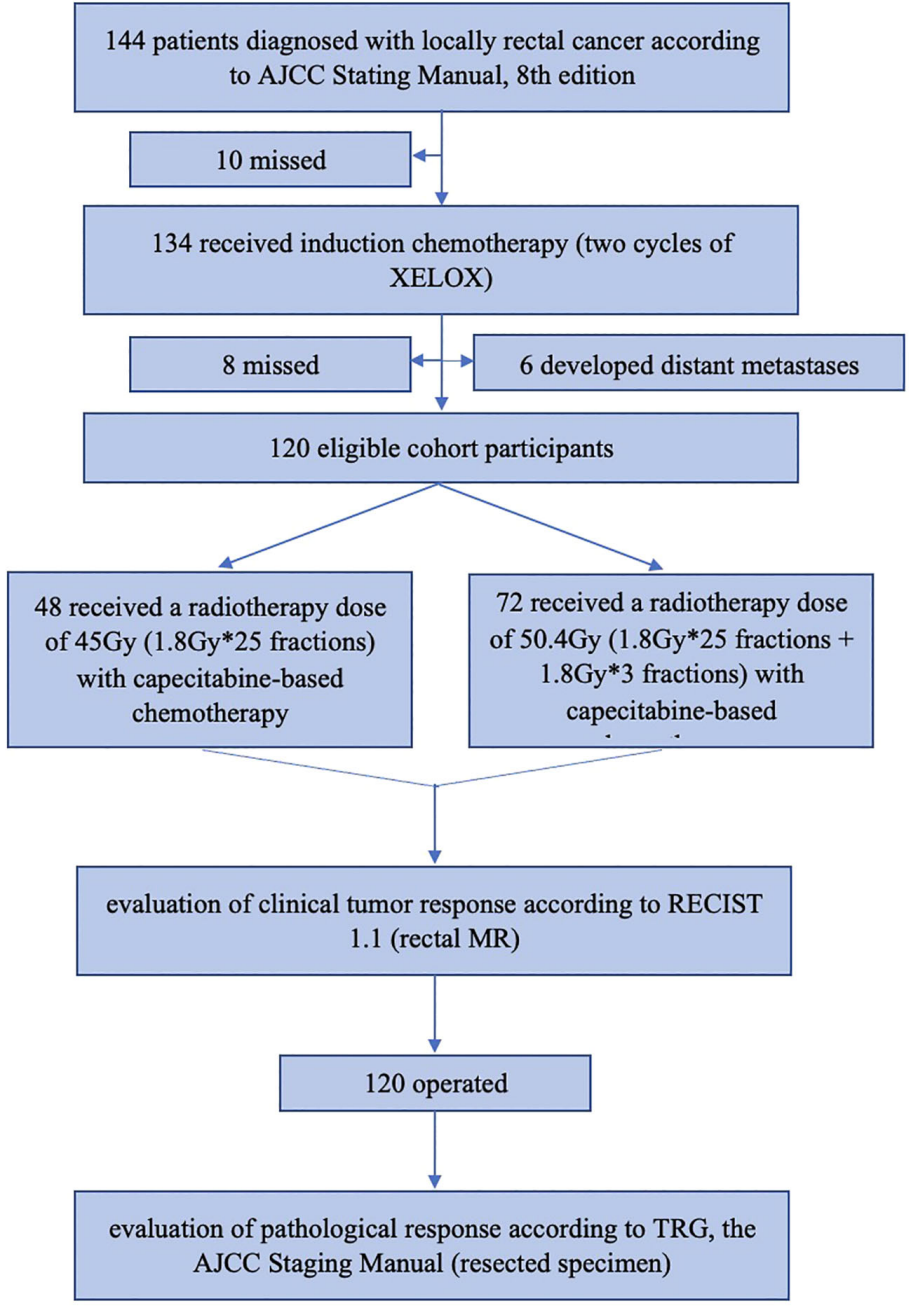
The patients flow diagram. AJCC, American Joint Committee on Cancer; XELOX regimen, Oxaliplatin in combination with capecitabine; MR, magnetic resonance.

144 patients were recruited in total, but 24 were excluded due to loss of follow-up or distant metastases before treatment, and 120 patients were enrolled eventually. The patient flow diagram is shown in **Figure 1**.

The inclusion criteria were as follows: (I) patients aged 18 to 75 years old; (II) rectal adenocarcinoma was diagnosed by colonoscopy; (III) Eastern Cooperative Oncology Group performance status (ECOG PS) was 0 or 1; (IV) defined as stage II/III according to the 8^th^ edition of the American Joint Committee on Cancer (AJCC) staging; (V) no distant metastases or concurrent malignancy; (VI) normal heart, liver and kidney function; (VII) underwent complete chemoradiotherapy and radical surgical treatment.

The exclusion criteria were as follows: (I) diagnosed with distant metastasis; (II) prior chemoradiotherapy or targeted therapy or immunotherapy; (III) patients with other malignancy and incomplete clinical data.

### Data collection

Patients baseline characteristics like age, gender, clinical stage, tumor location, tumor differentiation, circumferential resection margin (CRM) status, and extramural venous invasion (EMVI) status were collected on diagnosis. Adverse events, imaging assessments in pre- and post-nCRT, and pathological responses including epidermal growth factor receptor (EGFR) status, human epidermal growth factor receptor-2 (Her-2) status, and mismatch repair (MMR) status were recorded during follow-up observation. The time window for local recurrence rate, distant metastasis-free survival (DMFS), disease-free survival (DFS), and overall survival (OS) was from the date of surgery to the date of final follow-up. The last follow-up date was in November 2021. This study employed the outpatient system, the inpatient system, and telephone consultations to collect accurate patient information and to check for gaps through various collection methods. Regarding tumor location, tumors less than 5cm from the anus were considered low, tumors between 5-10cm from the anus were considered median, and tumors 10-12cm from the anus were considered high. The research was approved by the local ethics committee of The First Affiliated Hospital of Wenzhou Medical University and the Hospital Reviewing Board.

### Radiotherapy

Intensity-modulated radiation (IMRT) technology with the Elekta Synergy system (Elekta AB, Stockholm, Sweden) was utilized in this study. All patients were in the supine position. The CT scan range was from the upper boundary of the 2-3 lumbar vertebrae to the lower boundary of the upper 1/3 of the femur, with a thickness of 5mm. Contrast-enhanced venography was recommended if there were no contraindications. Calibration radiographs were taken for each patient at the first session and at regular intervals (once a week).

Radiotherapy targets for both groups (45Gy and 50.4Gy) were performed with the Monaco planning system according to the Radiation Therapy Oncology Group (RTOG) criteria. All patients were treated with 6 MV-X-rays. The specific target areas are outlined below. The gross tumor volume (GTV) was radiographically identified as gross lesions, including primary and metastatic lymph nodes. Clinical target volume (CTV) was defined as GTV + selective lymph node drainage area. CTV included the rectum and mesangial region, the presacral region, the internal iliac lymph nodes, and some obturator lymph nodes. The external iliac lymph nodes need to be irradiated when the tumor invades the bladder, prostate, and gynecological organs, and the external iliac and inguinal lymph nodes need to be irradiated when the tumor invades the anal canal or the lower 1/3 vagina. The upper boundary was the bifurcation of the common iliac artery, the lower boundary included the whole mesentery and was at least 2cm away from the lower edge of the tumor, the left and the right boundary was the inner edge of the true pelvis, the anterior boundary was 1cm in front of the posterior wall of the bladder or the anterior wall of the rectal organ; the internal iliac artery and vein were expanded by 0.7cm, and the posterior boundary was the front edge of the sacrum. Planning target volume (PTV) was defined as CTV+0.5-1.0cm.

The prescribed doses of PTV in the two groups were 45Gy and 50.4Gy, respectively. Patients in the 45Gy group received a total pelvic irradiation dose of 25×1.8Gy. Patients in the 50.4Gy group received a total pelvic irradiation dose of 45Gy (25×1.8Gy), and then the field was reduced to the mesenteric region for a supplement dose of 5.4Gy (3×1.8Gy). It should be pointed out that at least 95% of PTV received the specified dose. Radiotherapy was administered five times a week, from Monday to Friday. Organs at risk (OAR) mainly consisted of the femoral head, bladder and small intestine, the limited doses for each organ were as follows: femoral head Dmax <45Gy, bladder V50 <50Gy, and small intestine Dmax <50Gy.

### Chemotherapy regimens

XELOX- oxaliplatin at 135 mg/m^2^ and capecitabine at 1,000 mg/m^2^-was administrated twice a day for 14 days, every 21 days for 2 cycles before chemoradiotherapy. Based on several clinical studies like STAR-01, ACCORD, NSABP R-04, and PETACC 6, the addition of oxaliplatin to capecitabine-based chemoradiotherapy failed to improve the rates of pathological complete response(pCR) and OS as expected. Furthermore, it could increase grade 3/4 side effects, thereby affecting patient tolerance (29–31). According to the results of the ACCORD trial, there was no significant difference between the groups in terms of 3-year local recurrence (4%, 6%), DFS (74%, 69%), and OS (both 88%). Fluorouracil-based chemotherapy is still considered to be the first-line regimen during radiotherapy in LARC patients. Therefore, capecitabine was given simultaneously during radiotherapy, twice a day, on weekdays. Patients were assessed 5-12 weeks following nCRT, and surgery was performed.

### Adverse reactions monitoring

A variety of nCRT-related adverse reactions were evaluated, including bone marrow suppression, radioactive proctitis, intestinal obstruction or perforation, narrow lumen, anastomotic fistula, perianal skin injury, emesis, and hand-foot syndrome. Hand-foot syndrome was mainly associated with capecitabine treatment. During concurrent chemoradiotherapy, blood routine examinations and biochemical examinations were conducted weekly. RTOG radiation injury classification and Common Terminology Criteria for Adverse Events (CTCAE, Version 5) were adopted to assess adverse events. Grades 1 and 2 myelosuppression were considered mild, while grades 3 and 4 were considered moderate to severe. Similarly, grades 1 and 2 were defined as mild radiation proctitis, and grades 3 and 4 were defined as moderate to severe radiation proctitis. The remaining adverse reactions including intestinal obstruction or perforation, narrow lumen, anastomotic fistula, perianal skin injury, emesis, and hand-foot syndrome were evaluated by their occurrence or not.

### Therapeutic effect evaluation

Clinical tumor response was determined by senior radiologists using rectal magnetic resonance (MR) imaging after nCRT and in keeping with RECIST 1.1. Complete response(CR) or clinical complete response (cCR) is defined as the disappearance of all targets lesions; partial response(PR) is achieved when the sum of the target diameter is reduced by at least 30% from baseline; progressive disease (PD) is characterized by an increase of at 20% in minimum diameter of all target lesions, while stable disease (SD) is the state between PR and PD. Disease control rate (DCR) is defined as a radiographic assessment of CR, PR, and SD. Postoperatively, the efficacy was evaluated by the pathological response. Pathological tumor response was evaluated by two experienced pathologists using resected specimens after TME and in accordance with TRG (the AJCC Staging Manual), with the four-tier AJCC Staging Manual being our study’s preferred evaluation method (32). Grade 0-pCR- is complete regression with the absence of cancer cells; grade 1 is moderate regression with single or few cancer cells remaining; grade 2 is mild regression and surplus tumor with extensive fibrotic stroma; grade 3 is no regression and extensive tumor residue accompanied by no or little tumor cell necrosis. Grades 0 and 1 are considered good pathological regression, while grades 2 and 3 are considered poor pathological regression.

### Statistical analysis

SPSS 23.0 software (USA) was used for statistical analysis. Age, gender, clinical stage, tumor location, tumor differentiation, imaging reports, pathological response, imaging assessment, anal retention rate, disease control rate, and adverse events were compared using the χ2 test for the two groups. Univariate and multivariate Cox regression analyses were conducted to identify characteristics that related to survival in patients. Age, gender, clinical stage, tumor location, tumor differentiation, CRM status, EMVI status, EGFR status, MMR status, Her-2 status, and radiation dose were the included variable. The Kaplan-Meier method was used to estimate the survival curves, and the log-rank test was used for comparative analysis. A P-value<0.05 was considered statistically significant.

## Results

### Baseline characteristics

The baseline characteristics are shown in **Table 1**. The 45Gy group consisted of patients with a median age of 59.5years (from 36 to79 years) while the 50.4Gy group consisted of patients with a median age of 58 years (from 38 to 77 years), with a male predominance in both groups. No significant difference was observed between the 50.4Gy group and the 45Gy group in terms of clinical stage, tumor location, tumor differentiation, and several biological features.

**Table 1:** Baseline characteristics of patients.

Characteristics	45Gy group	50.4Gy group	χ2	p
Age (years)			0.051	0.821
Median (Range)	59.5 (36-79)	58 (38-77)		
>55	27	42		
≤55	21	30		
Gender			0.068	0.794
Male	37	54		
Female	11	18		
Clinical stage				
T3	36	51	0.251	0.617
T4	12	21		
N0	6	8	1.001	0.606
N1	17	20		
N2	25	44		
Tumor location			1.066	0.587
Low	22	27		
Mid	25	42		
High	1	3		
Tumor differentiation			0.988	0.610
Poorly differentiated	9	12		
Moderately differentiated	23	41		
Well differentiated	16	19		
CRM			1.442	0.230
(+)	18	35		
(-)	30	37		
EMVI			0.050	0.823
(+)	23	33		
(-)	25	39		
EGFR			4.300	0.116
(+)	24	46		
(-)	13	19		
N/A	11	7		
Her-2			3.952	0.157
(+)	9	15		
(-)	28	50		
N/A	11	7		
MMR			4.263	0.122
pMMR	29	54		
dMMR	8	11		
N/A	11	7		

CRM, circumferential resection margin; EMVI, extramural venous invasion; EGFR, epidermal growth factor receptor; Her-2, human epidermal growth factor receptor-2; MMR, mismatch repair; dMMR, mismatch-repair-deficient; pMMR, mismatch-repair-proficient; N/A, not applicable.

### Treatment outcomes

**Figure 2:**
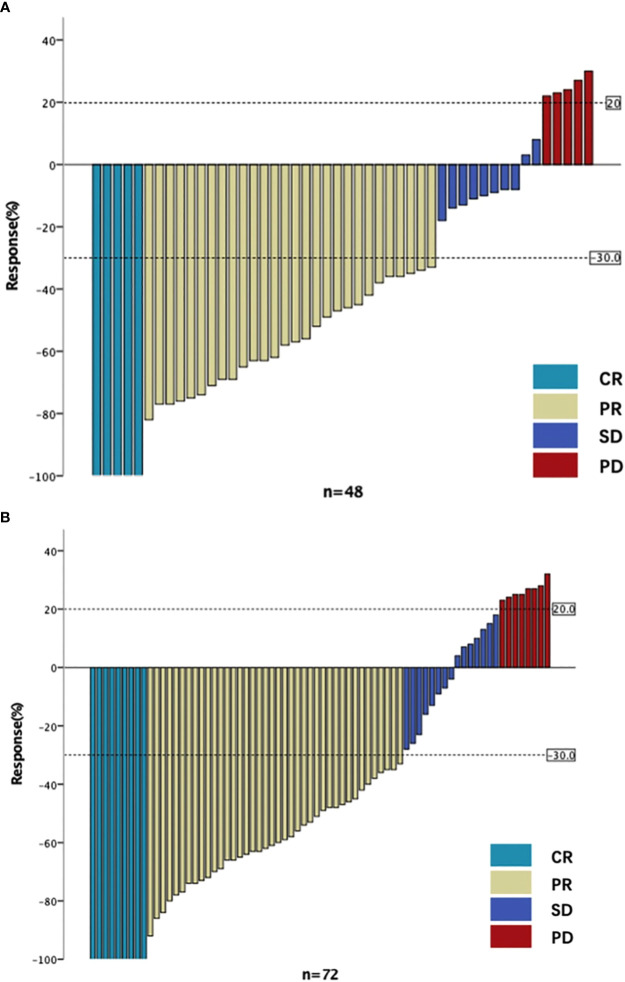
The imaging evaluation in two groups. **(A)** The imaging evaluation in 45Gy group. **(B)** The imaging evaluation in 50.4Gy group. CR, complete response; PR, partial response; SD, stable disease; PD, progressive disease.

The rate of good pathological response (grade 0/1) was 59.72% in the 50.4Gy group (43/72), while it was 64.58% in the 45Gy group (31/48). The DCR in the 50.4Gy group was 91.67% (66/72), compared to 89.58% in the 45Gy group (43/48). The anal preservation rate in the 50.4Gy group was 79.17% (57/72), compared to 60.42% (29/48) in the 45Gy group (P<0.05). The efficacy of nCRT is shown in **Table 2**. Imaging evaluation in pre- and post-nCRT for patients is shown in **Figure 2**.

**Table 2:** Efficacy evaluation of nCRT.

Characteristics	45Gy group	50.4Gy group	χ2	p
TRG stage			0.288	0.592
Grade 0,1	31	43		
Grade 2,3	17	29		
Imaging evaluation			0.005	0.945
DCR (CR+PR+SD)	43	64		
PD	5	8		
Operation			4.986	0.026
Anal-preservation	29	57		
Non-anal-preservation	19	15		

DCR, disease control rate; CR, complete response; PR, partial response; SD, disease stability; PD, disease progression; nCRT, neoadjuvant chemoradiotherapy.

As demonstrated in **Table 3**, the local recurrence rate in the 50.4Gy group was 6.94% (5/72), the distant metastasis rate was 18.06% (13/72), and the DCR was 88.89% (64/72), compared with 4.17% (2/48), 22.92% (11/48), and 89.58% (43/48) in the 45Gy group, respectively. No statistical difference was found between the two groups.

**Table 3:** Disease control situation.

Control condition	45Gy group	50.4Gy group	χ2	p
Local recurrence rate (%)	4.17 (2/48)	6.94 (5/72)	0.057	0.811
Distant metastasis rate (%)	22.92 (11/48)	18.06 (13/72)	0.425	0.514
Disease control rate (%)	89.58 (43/48)	91.67 (66/72)	0.151	0.698

Univariate Cox regression analysis showed that tumor differentiation, Her-2 status, and MMR status were associated with DFS, HR=0.312 (tumor differentiated), 0.505 (Her-2), 0.344(MMR) (tumor differentiated 95% CI: 0.176–0.555, Her-2 95% CI: 0.263–0.968, MMR 95% CI: 0.147–0.804). Multivariate Cox regression analysis implied that tumor differentiation was an independent predictor for DFS (see **Table 4**). Univariate and multivariate Cox regression analysis revealed that only tumor differentiation was closely related to OS, HR=0.232 (95% CI: 0.068-0.794). However, Cox regression analysis showed that the radiation dose was not an independent predictor for DFS (HR=1.118, 95% CI: 0.559-2.525) or OS (HR=1.321, 95% CI: 0.293-5.945). Notably, the base variables of univariate and multivariate Cox regression analyses have been bolded and skewed in **Tables 4**, **5**.

**Table 4:** Univariate and multivariate Cox regression analyses for different variables and DFS in LARC patients.

Variables	Disease free survival (n=120)			
	Univariate analysis		Multivariate analysis	
	HR (95% CI)	p	HR (95% CI)	p
Age (≤55 vs. ***>55years***)	3.120 (0.941-10.343)	0.063		
Gender (***male*** vs. female)	0.876 (0.413-1.859)	0.730		
Clinical stage (***T3*** vs. T4)	1.394 (0.642-3.024)	0.401		
Clinical stage (***N0*** vs. N1 vs. N2)	1.111 (0.645-1.912)	0.704		
Tumor location (***low*** vs. mid vs. high)	1.050 (0.529-2.087)	0.888		
Tumor differentiated (***poorly*** vs. moderately vs. well)	0.312 (0.176-0.555)	<0.001	0.380 (0.204-0.708)	0.002
CRM (***positive*** vs. negative)	0.703 (0.334-1.479)	0.353		
EMVI (***positive*** vs. negative)	0.695 (0.330-1.464)	0.339		
EGFR (positive vs. ***negative*** vs. N/A)	0.588 (0.335-1.031)	0.064		
MMR (***pMMR*** vs. dMMR vs. N/A)	0.344 (0.147-0.804)	0.014	1.246 (0.494-3.147)	0.641
Her-2 (positive vs. ***negative*** vs. N/A)	0.505 (0.263-0.968)	0.040	0.427 (0.147-1.244)	0.119
Radiation dose (***45Gy*** vs. 50.4Gy)	1.118 (0.559-2.525)	0.655		

DFS, disease-free survival; LARC, locally advanced rectal cancer; CRM, circumferential resection margin; EMVI, extramural venous invasion; EGFR, epidermal growth factor receptor; MMR, mismatch repair; dMMR, mismatch-repair-deficient; pMMR, mismatch-repair-proficient; N/A, not applicable; Her-2, human epidermal growth factor receptor-2.

**Table 5:** Univariate and multivariate Cox regression analyses for different variables and OS in LARC patients.

Variables	Overall survival (n=120)			
	Univariate analysis		Multivariate analysis	
	HR (95% CI)	p	HR (95% CI)	p
Age (≤55 vs. ***>55years***)	4.778 (0.570-40.067)	0.149		
Gender (***male*** vs. female)	0.030 (0.000-36.221)	0.334		
Clinical stage (***T3*** vs. T4)	1.941 (0.430-8.756)	0.388		
Clinical stage (***N0*** vs. N1 vs. N2)	1.345 (0.426-4.245)	0.614		
Tumor location (***low*** vs. mid vs. high)	1.675 (0.454-6.178)	0.439		
Tumor differentiated (***poorly*** vs. moderately vs. well)	0.232 (0.068-0.794)	0.020	0.232 (0.068-0.794)	0.020
CRM (***positive*** vs. negative)	0.256 (0.050-1.326)	0.105		
EMVI (***positive*** vs. negative)	0.298 (0.058-1.538)	0.148		
EGFR (positive vs. ***negative*** vs. N/A)	0.457 (0.138-1.515)	0.200		
MMR (***pMMR*** vs. dMMRvs. N/A)	0.327 (0.057-1.888)	0.211		
Her-2 (positive vs. ***negative*** vs. N/A)	0.356 (0.064-1.979)	0.238		
Radiation dose (***45Gy*** vs. 50.4Gy)	1.321 (0.293-5.945)	0.717		

OS, overall survival; LARC, locally advanced rectal cancer; CRM, circumferential resection margin; EMVI, extramural venous invasion; EGFR, epidermal growth factor receptor; MMR, mismatch repair; dMMR, mismatch-repair-deficient; pMMR, mismatch-repair-proficient; N/A, not applicable; Her-2, human epidermal growth factor receptor-2.

**Figure 3:**
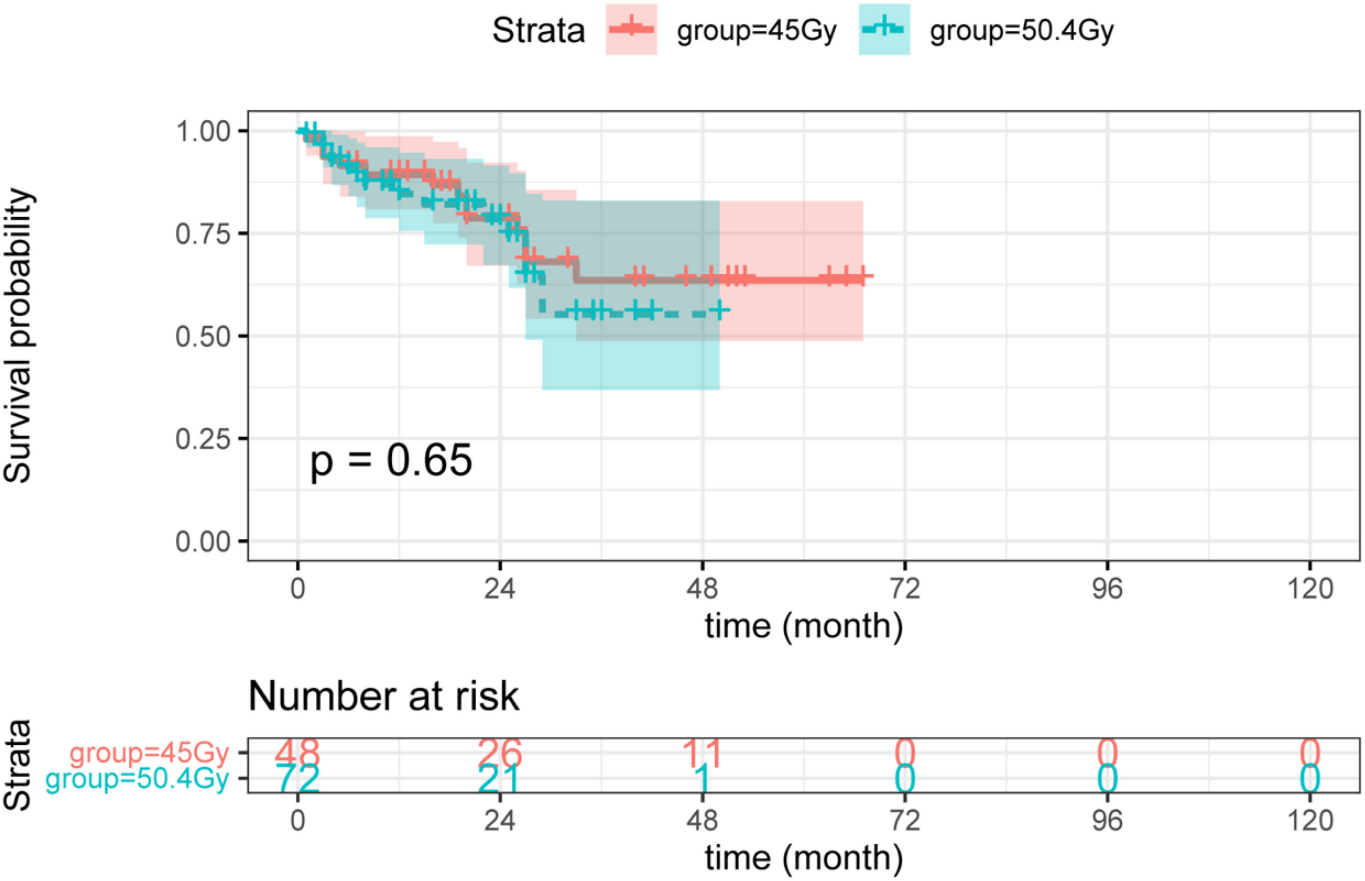
The DFS between LARC patients with different radiation doses. DFS, disease-free survival; LARC, locally advanced rectal cancer.

**Figure 4:**
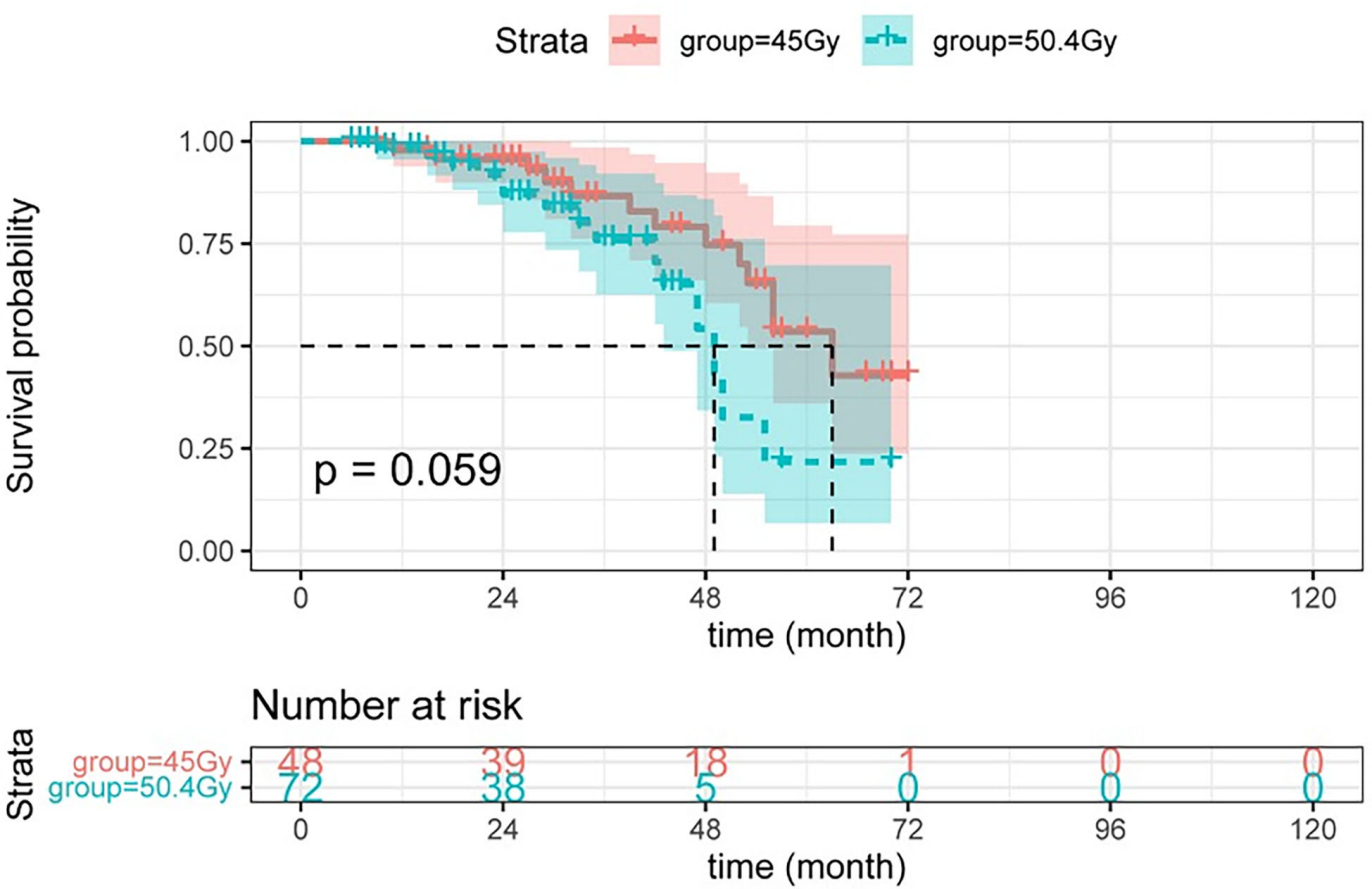
The OS between LARC patients with different radiation doses. OS, overall survival; LARC, locally advanced rectal cancer.

Herein, higher radiation doses did not confer longer DFS (see **Figure 3**). As shown in **Figure 4**, there was no statistical difference between the two groups regarding OS. Radiotherapy dose intensification was not significant in this study, the long-term survival outcomes in the 45Gy group were comparable to outcomes in the 50.4Gy group.

### Adverse reactions

CTCAE assessment of myelosuppression and radiation proctitis was evaluated by the RTOG radiation injury classification. The incidence of myelosuppression, radiation proctitis, and intestinal obstruction or perforation in the high-dose group was higher than that in the low-dose group, and the differences were statistically significant. Incidence of narrow lumen, anastomotic fistula, perianal skin injury, emesis, and the hand-foot syndrome showed no significant difference between the two groups. The incidence of specific adverse events is listed in **Table 6**.

**Table 6:** Adverse reactions to treatment.

Characteristics	45Gy group	50.4Gy group	χ2	p
Radioactive proctitis			6.699	0.010
Grade 1,2	43	50		
Grade 3,4	5	22		
Myelosuppression			5.412	0.020
Grade 1,2	32	41		
Grade 3,4	7	27		
Intestinal obstruction or perforation	1	11	4.201	0.040
Narrow lumen	2	4	0.119	0.730
Anastomotic fistula	5	12	0.925	0.336
Skin lesions around the anus	17	24	0.056	0.814
Emesis	15	24	0.057	0.811
Hand-foot syndrome	8	13	0.038	0.844

## Discussion

In our retrospective study, we found that the survival outcomes of LARC patients treated with 50.4Gy were similar to those of patients treated with 45Gy. In the 50.4Gy group, the rates of pCR and cCR were 19.4% (14 of 72), and 12.5% (9 of 72), respectively, while in the 45Gy group, the rates were 22.9% (11 of 48), and 10.4% (5 of 48), showing no statistical significance. After radiotherapy dose transmutation from 45Gy to 50.4Gy, we observed no improvement in the rate of pCR, while there was a slight improvement in the rate of cCR. The rate of cCR in our study in question was partly based on data from a randomized phase 2 trial, where the rate of clinical complete/near-complete tumor response at MR did not increase after dose escalation from 50Gy to 65Gy (27). Radiotherapy dose intensification was not significant in this study. The statistics from a randomized trial showed that brachytherapy boost supplementation to conventional radiotherapy dose could improve the rate of near-complete response, but not that of pCR (33). Previously reported dose-response relationships may largely be directed by grade 0 and grade 1 (TRG, the AJCC Staging Manual), which could partly explain the rates of pCR in our study.

A radiotherapy dose of 50.4Gy was associated with higher rates of adverse reactions such as radioactive proctitis, myelosuppression, and intestinal obstruction or perforation. In the 45Gy group, 2.1% (1 of 48) of patients experienced intestinal obstruction, while 6.9% (5 of 72) of patients had intestinal obstruction, and 8.3% (6 of 72) had intestinal perforation in the 50.4Gy group. Due to a significant disadvantage relative to the rate of radioactive proctitis, the intestinal perforation rate was higher in the 50.4Gy group than in the 45Gy group. Among the patients in the 45Gy group, 14.6% (7 of 48) had severe myelosuppression compared with 37.5% (27 of 72) in the 50.4Gy (P<0.05). The districts of radiotherapy for LARC generally include the pelvis and pelvic lymph node areas, which exposes to hematological toxicity in the range of 30%-70% and there is a dose-likelihood efficiency (34, 35).

Colorectal cancer is an intractable worldwide public health issue due to its huge disease burden. The prevalence of western lifestyles, dietary changes, and reduced physical activity are the main reasons for the continued rise in colorectal cancer incidence worldwide (36). Multidisciplinary-based treatment is strongly recommended since advancements in diagnostic imaging and an evidence-based combination of chemotherapy, radiotherapy, and TME can markedly improve the prognosis of LARC patients. Especially in the case of a resectable lesion, the integration of chemotherapy and radiotherapy can achieve favorable tumor downstaging and local control rate (37, 38). However, owing to metastasis to other organs or local recurrence, the long-term survival of LARC is unsatisfactory (4, 17). To achieve a better prognosis, imaging (39, 40), carcinoembryonic antigen (CEA) combined with carbohydrate antigen 19-9 (CA19-9) (41), platelet-associated biomarkers (42), and circulating tumor DNA (ctDNA) (43) must be dynamically estimated and promptly evaluated. A more personalized treatment regimen is preferable for high risk patients, and novel combination regimens should be further investigated.

SCRT, which is the conventional treatment in European countries, is developing rapidly. Generally, patients receive pelvic radiotherapy at a dose of 5×5Gy during the first week, followed by surgical intervention and six sessions of adjuvant chemotherapy. Interestingly, no significant difference in recurrence rates, distant metastasis, or late adverse events compared to long-term radiotherapy was observed (44). Moreover, a single-arm phase II clinical research (45) concluded that SCRT followed by chemotherapy plus immunotherapy and surgery demonstrated an impressive pCR rate with good tolerance in patients. Furthermore, SCRT treatment resulted in fewer late adverse events and rectal injury (46, 47). However, another randomized trial concluded that SCRT with delayed surgery was associated with an increased risk of local recurrence after a 10-year follow-up period (48).

Given the association between treatment intensification-tumor response and tumor prognosis, more consolidated treatment options are needed. TNT-chemotherapy combined with chemoradiotherapy before surgery-is a novel treatment approach for LARC patients, achieving improved downstaging, patient compliance, and micrometastases elimination rate (49–51). TNT is a promising systemic strategy to target micrometastases, especially for patients unfit for surgery (49). The pCR rate in patients treated with TNT (36%) was found to be higher compared to patients receiving nCRT (21%) (49). Patients who achieved pCR may choose non-operative treatment, sphincter-sparing surgery, or observation and periodic review. Nowadays, chemotherapy is administrated before or after radiotherapy, and the sequence of radiotherapy, chemotherapy and surgery has been extensively explored. In general, INCT combined with CRT is a preferred method since it is associated with better compliance and fewer acute adverse reactions (13, 50, 52). In our study, all patients underwent two cycles of induction chemotherapy and fluorouracil-based chemoradiotherapy.

Cancer is more averse to becoming a “chronic disease” as medical technology advances and therapeutic methods evolve. Functional needs and survival needs (46) are two central issues we should aim to address in future treatment prospects of rectal cancer. Our goal at the moment is to achieve a complete resection of the lesion while sparing the functioning sphincter complex of the anus, thereby improving the quality of life of patients. Notably, surgical improvements have helped reduce the local recurrence rate from above 50% to below 10% (53, 54). Herein, we noticed a significantly higher rate of anal retention in the 50.4Gy group compared to the 45Gy group (79.2% (57 of 72) vs. 60.4% (29 of 48)).

CRM and EMVI are important factors that predict survival outcomes and contribute to clinical treatment planning (55, 56). As a result, no significant difference between the two groups was observed. The survival curve in this study was not statistically significant, but the 45Gy group had a higher 80-month survival rate, which may be related to the higher incidence of adverse reactions in the 50.4Gy group, particularly intestinal obstruction or perforation and myelosuppression. Furthermore, according to our findings, a radiotherapy dose of 50.4Gy resulted in a favorable anal retention rate but at the expense of increased rates of several adverse reactions, with no improvement in the rate of good pathological response, DFS or OS.

Nevertheless, our study has several limitations. We included a small number of patients from a single center, and the follow-up time was not long enough to obtain long-term survival statistics. Moreover, our study was retrospective in nature, which may have resulted in bias to some extent. Nonetheless, to our knowledge, this is the first study that concluded the rate of pCR in the 45Gy group was higher than that of the 50.4Gy group. This study has clinical guiding significance and, to some extent, provides the basis for choosing radiotherapy doses in LARC patients. A radiotherapy dose of 50.4Gy is preferred for a higher likelihood of anal retention if a patient-centered outcome is prioritized. Meanwhile, if an efficacy-centered outcome is preferred, a radiotherapy dose of 45Gy is desired for a greater degree of pCR and a lower likelihood of adverse reactions. Combined with the trend of individualized treatment of the tumor, the radiotherapy dose needs to be considered according to the tolerance of the patient, which includes age, ECOG PS, and underlying disease. In the follow-up treatment, the efficacy and quality of life are the focus of doctors.

## Conclusion

A radiation dose of 50.4Gy contributes to a better anal retention rate but at the cost of serious adverse events and failure to improve the rate of good pathological response, imaging remission, DFS or OS.

## Data availability statement

The raw data supporting the conclusions of this article will be made available by the authors, without undue reservation.

## Author contributions

(I) Conception and design: XZ, CZ; (II) Administrative support: HZ; (III) Provision of study materials or patients: YX, ZS; (IV) Collection and assembly of data: All authors; (V) Data analysis and interpretation: XR; (VI) Manuscript writing: All authors. (VII) All authors contributed to the article and approved the submitted version.
